# The Anti-Inflammatory Effect of *Smilax china* L. Extract on LPS-Stimulated THP-1 via Downregulation of MAPK and NF-*κ*B Signaling Pathway

**DOI:** 10.1155/2021/9958808

**Published:** 2021-11-16

**Authors:** Siyi Jiang, Qiong Wei, Xiaochuan Ye, Dan Luo, Xiaoyan Zhang, Zhenglei Li, Pengtao You, Xianzhang Huang, Yanwen Liu

**Affiliations:** ^1^College of Pharmacy of Hubei University of Chinese Medicine, Wuhan, Hubei 430061, China; ^2^Hubei Province Key Laboratory of Traditional Chinese Medicine Resource and Chemistry, Wuhan, Hubei 430061, China; ^3^Henan Key Laboratory of Zhang Zhongjing Formulae and Herbs for Immunoregulation, Nanyang Institute of Technology, Nanyang 473004, China

## Abstract

**Background:**

Traditional Chinese medicine Smilax is the rhizome of liliaceous plant *Smilax china* L., which is used to treat pelvic inflammatory disease and anxieties.

**Purpose:**

To investigate the mechanism of anti-inflammatory activity of the extract from *Smilax china* L. (ES).

**Methods:**

The components of ES were identified by UPLC-QTOF-MS/MS. The anti-inflammatory activities were evaluated in xylene-induced ear oedema and egg white-induced plantar swelling test. Cell viability was examined by CCK-8 assay. The inflammatory mediators, proinflammatory cytokines, and MAPK and NF-*κ*B signals in LPS-stimulated THP-1 cells were determined using ELISA, real-time PCR, and Western blot, respectively.

**Results:**

20 compounds of ES were confirmed by comparing with the reference substance. ES displayed more prominent anti-inflammatory activity than the positive control “Jin Gang Teng” capsule in the *in vivo* acute inflammatory model. ES suppressed the expression of PGE_2_ and 6-Keot-PGF_1_*α*, and the ratio of IC_50_ (COX-1)/IC_50_ (COX-2) of ES was 3.15, which indicated that ES could selectively inhibit COX-2. ES dose-dependently (12.5, 25, and 50 mg/L) decreased the production and mRNA levels of proinflammatory cytokines IL-1*β*, IL-6, and TNF-*α*. Furthermore, ES significantly decreased LPS-induced phosphorylation of p38, JNK, ERK1/2, and p65, inhibiting the expression of IKK*α* and the degradation of I*κ*B*α*.

**Conclusion:**

The results suggested that ES could selectively inhibit the activity of COX-2, and the anti-inflammatory effect of ES was associated with the inhibition of IL-1*β*, IL-6, and TNF-*α* via negative regulation of MAPK and NF-*κ*B signaling pathways in LPS-induced THP-1 cells.

## 1. Introduction

Inflammation is the first response occurring after damage or infection, which was regulated by many inflammatory mediators, including cyclooxygenase-2 (COX-2), prostaglandin E2 (PGE_2_), and proinflammatory cytokines (IL-1*β*, IL-6, and TNF-*α*) [1–3]. It has been generally accepted that the excessive production of proinflammatory cytokines and mediators due to monocytes and macrophages activation plays an important role in the progression of many inflammatory disease, such as rheumatic arthritis, chronic bronchitis, colitis, and glomerulonephritis [4]. During the inflammatory condition, monocytes and macrophages are activated; they secrete massive proinflammatory cytokines to regulate immune responses. Simultaneously, a large amount of PGE_2_ is generated by the inducible proteins COX-2, which induces the body's response to pain and inflammation [5].

LPS-induced THP-1 cells have been widely used as an *in vitro* model because THP-1 which is a human leukemia monocytic cell line can be stimulated by LPS to trigger the activation of multiple inflammatory signals such as mitogen-activated protein kinases (MAPKs) and nuclear transcription factor kappa-B (NF-*κ*B) [6, 7]. MAPKs containing p38 MAPK, ERK, and JNK have important functions on regulation of cell differentiation, cell growth, and cellular response to cytokines in the immune system [8]. NF-*κ*B signaling pathway plays a crucial role in regulating inflammation through transcription of COX and cytokine genes [9]. NF-*κ*B is normally located in the cytoplasm with its inhibitor I*κ*B*α*. However, LPS stimulation induces phosphorylation of I*κ*B*α* leading to translocation of NF-*κ*B into the nucleus where it activates transcription [10]. The activation of MAPK and NF-*κ*B triggers the expression of genes encoding downstream inflammatory mediators and eventually causes inflammatory actions [11]. *Smilax china* L., also known as “Jin Gang Teng,” belongs to Liliaceae family. As a commonly used traditional Chinese medicine, the herb has been collected in the Chinese Pharmacopoeia (2015 version) and generally used to treat pelvic inflammatory disease, adnexitis, lump, and other diseases of gynecology clinically [12]. The efficacy of *Smilax china* L. has been declared on anti-inflammation [13], antinociception [14], and anticancer [15]. “Jin Gang Teng” capsule made from water extract of the herb has a good effect on gynecological inflammation, and its annual sales exceed 100 million. But the extraction rate of active components in water extract is low, and the mechanism of action is not entirely clear. The herb contains many chemical ingredients such as flavonoids, saponins, stilbene glycosides, polyphenols, etc. Previous research results showed that the flavonoids, saponins, and polyphenols of the herb are active ingredients inhibiting inflammation [16]. Our research group prepared extract using ethanol extraction and macroporous adsorption resin purification and improved the extraction and concentration rate of chemical compositions including flavonoids, saponins, tannins, etc. Although it has been reported that the herb can decrease the production of PGE_2_ and inhibit COX-2 activity [13], its anti-inflammatory mechanisms as well as the associated signaling pathways have not yet been fully illuminated. Therefore, in the present study, the components of ES were identified by UPLC-QTOF-MS/MS, and anti-inflammatory mechanisms of ES were investigated in LPS-induced THP-1 cells.

## 2. Materials and Methods

### 2.1. Preparation of ES

The dried powdered rhizomes of *Smilax china* L. (100 g) were firstly treated with 800 ml 60% ethanol 2 hours and subsequently with 600 ml 60% ethanol 1 hour for two more times. The extracts of the three treatments were collected, concentrated under reduced pressure, and filtered to get the total extracts. 70 ml 3% gelatin solution was added to the total extracts and placed statically for 24 h and then filtered. Distilled water was added to get filtrate at the concentration of 0.2 g/ml, which was subjected to D101 macroporous adsorption resin (2 BV/h) and successively eluted with 3 BV distilled water (2 BV/h) until the Molisch reaction was negative and then successively eluted with 5 BV 70% ethanol (2 BV/h). The eluate of 70% ethanol was collected and dried by rotary evaporation to obtain ES.

### 2.2. Chromatography

ES (0.05 g) was sonicated in 25 mL of methanol for 45 min. Prior to injection, an adequate volume (2 mL) was passed through a 0.22 *μ*m membrane filter. A UPLC-QTOF-MS/MS system (Aglient, USA) with ZORBAX RRHD SB C18 column (100 mm × 2.1 mm, 1.8 mm) was used to detect the main compounds of ES. The following conditions were used for detection: column temperature 40°C, flow rate 0.4 mL/min, detection wavelength 290 nm (0–25 min) and 370 nm (25–30 min), mobile phase (A) methanol: acetonitrile (5 : 1), (B) 0.1% formic acid, and elution program is shown in Table 1. The injection volume was 1 *μ*L. Mass spectrometry was used with the following conditions: sheath gas velocity 8 L per min, temperature 320°C, voltage 3.0 kV, negative ion mode, and range of measurement m/z = 100–1000.

### 2.3. *In Vivo* Anti-Inflammatory Activity Determination

The animal experiments were complied with the Guide for the Care and Use of Laboratory Animals (NIH Publication no. 85–23, revised 1996) and approved by the Committee of Hubei University of Chinese Medicine for Institutional Animal Care and Use. Female Wistar rats (200 ± 20 g) and female Kunming mice (18 ± 2 g) were purchased from the Hubei experimental animal center (China, License no. SCXK (E) 2008–0005). They were housed in animal rooms under standard condition of light and temperature with a 12 h/12 h light/dark cycle.

The positive control “Jin Gang Teng” capsules (JGT) were purchased from Hubei Furen Pharmaceutical Co., Ltd. (Batch no. 090329).

#### 2.3.1. Xylene-Induced Ear Oedema

Forty female Kunming mice were randomly divided into four groups: model group (saline), JGT group (the extract at the dose equalling to 13.5 g herb/kg), and two doses of ES (the extract at the doses equalling to 13.5 and 27 g herb/kg). Four groups were administered orally once a day for 4 days. 1 h after the last administration, the left ear was coated with 0.1 mL xylene in all mice and the right ear was considered as control. After 15 min, the animals were sacrificed, the ears were cut off and weighed. The swelling degree of ear was calculated using formula: degree of era oedema (%) = (the weight of the left ear − the weight of the right ear)/the weight of the right ear × 100%; inhibiting rate of inflammation (%) = mean of model group − mean of test group.

#### 2.3.2. Egg White-Induced Plantar Swelling

Forty female Wistar rats were randomly divided into four groups: model group (saline), JGT group (the extract at the dose equalling to 10 g herb/kg), and two doses of ES (the extract at the doses equalling to 10 and 20 g herb/kg). Four groups were administered orally once a day for 14 days. On the 14th day, the volume of left hind paw of rats was measured. 1 h after the last administration, 0.05 mL of freshly prepared 10% fresh egg white aqueous solution was injected subcutaneously into the left hind paw of rats in each group. After 6 h, the volume of left hind paw was measured and the swelling rate of paw was calculated using formula: degree of plantar swelling (%) = (the volume of left hind paw − original volume of left hind paw)/original volume of left hind paw × 100%; inhibiting rate of inflammation (%) = mean of model group − mean of test group.

### 2.4. Cell Culture

THP-1 human monocytic cells from China Center for Type Culture Collection (CCTCC, Wuhan, China) were cultured in RPMI 1640 medium (Hyclone, USA) supplemented with 10% fetal bovine serum (FBS) (Gibco, USA), 100 U/mL penicillin, and 100 *μ*g/mL streptomycin. Cells were incubated in an incubator at 37°C with 95% air and 5% CO_2_.

### 2.5. Cell Viability

Cell viability was examined using cell counting kit (CCK-8) (Engreen Biosystem, BJ, China). Briefly, THP-1 cells were seeded into 96-well plates at a density of 2 × 10^5^ cells/mL. ES was added to the cells at the indicated concentrations (0, 6.25, 12.5, 25, 50, 100, 200, and 400 *μ*g/mL) and incubated at 37°C for 24 h. An amount of 10 *μ*L CCK-8 reagent was added to each well and incubated at 37°C for 4 h. Absorbance was measured at 450 nm using Model 680 microplate reader (Bio-Rad, CA, USA).

### 2.6. Determination of 6-Keot-PGF_1_*α* and PGE_2_ in LPS-Induced THP-1 Cells

THP-1 cells were seeded in 96-well plates at a density of 2 × 10^5^ cells/mL. Cells were pretreated with LPS (1 *μ*g/mL) for 12 h, then incubated with ES (6.25, 12.5, 25, 50, 100 *μ*g/mL) for 12 h, and finally added with AA (10 *μ*mol/L) for 30 min. Supernatant was collected and analyzed for cytokines production by using ELISA kits (Elabscience Biotechnology Co., Ltd., Wuhan, China) according to the manufacturer's protocols. Indomethacin (National Institutes for Food and Drug Control) and meloxicam (National Institutes for Food and Drug Control) were positive control. 6-Keot-PGF_1_*α* was measured as an indicator of COX-1 activity, while PGE_2_ was measured as an indicator of COX-2 activity. The inhibition percentage of 6-Keot-PGF_1_*α* and PGE_2_ was calculated and the half-maximal inhibitory concentration (IC_50_) values were determined by regression analysis.

### 2.7. Determination of IL-1*β*, IL-6, and TNF-*α* in LPS-Induced THP-1 Cells

THP-1 cells were seeded in 96-well plates at 2 × 10^5^ cells/mL. The cells were treated with LPS (1 *μ*g/mL) and ES (0, 6.25, 25, and 50 *μ*g/mL) for 12 h. Supernatant was collected and analyzed for cytokines production by using ELISA kits (Elabscience Biotechnology Co., Ltd., Wuhan, China) according to the manufacturer's protocols.

### 2.8. Real-Time PCR

Total RNA was extracted by Trizol RNA isolation reagent (Invitrogen). The cDNA was generated using the First-Strand cDNA Synthesis Kit (TOYOBO). The synthesized cDNA was amplified by using SYBR® Premix Ex Taq™ (Takara) and quantitative real-time PCR assays were carried out in a Bio-Rad CFX96 touch q-PCR system (Bio-Rad, USA). The primer sequences (Invitrogen Biotechnology Co., Ltd., China) used for IL-1*β*, IL-6, TNF-*α*, and GAPDH are shown in Table 2. The PCR reaction consisted of denaturation at 95°C for 1 min, followed by 40 cycles of 15 s at 95°C, 20 s at 58°C, and 20 s at 72°C. All signals were normalized with GAPDH and the mRNA relative expression was calculated according to the methods of 2^−△△Ct^ (where △△Ct = △Ct sample − △Ct reference).

### 2.9. Western Blot

THP-1 cells were seeded in 6-well plates at 1.5 × 10^6^ cells/mL. The cells were pretreated with or without LPS (1 *μ*g/mL) for 1 h and then treated with ES (0, 12.5, 25, and 50 *μ*g/mL) for 1 h. After incubation, the cells were harvested by centrifugation (1500 rpm, 5 min). Proteins were extracted by RIPA lysis Kit (Beyotime) containing phenylmethanesulfonyl fluoride (PMSF). Protein concentration in the supernatant was measured using BCA protein assay kit (Takara, Japan). Equal amounts of proteins were loaded on 10% SDS-polyacrylamide gel and transferred onto nitrocellulose membranes (Amersham Biosciences, UK). After blocking in Tris-buffered saline with 0.05% Tween 20 (TBST) containing 5% nonfat milk for 1.5 h, the membranes were incubated with primary antibodies (Cell Signaling Technology) at 4°C overnight. After washing, the membranes were incubated with HRP-conjugated IgG antibodies (Cell Signaling Technology) for 1 h. Membranes were developed using ECL Western blotting detection reagent (Bio-Rad). The band intensity was measured using the FluorChem 8000 system.

### 2.10. Statistical Analysis

The results were presented as means ± SD. Statistical significance was determined by ANOVA and Student's *t*-test using SPSS software (IBM SPSS Statistics version 19.0, IBM Company). Values of *P* < 0.05 were considered as statistically significant.

## 3. Results

### 3.1. UPLC-QTOF-MS/MS Analysis of ES

**Figure 1:**
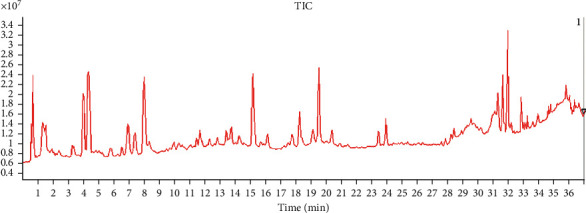
Total ion chromatogram (TIC) of ES detected by UPLC-QTOF-MS/MS.

58 compounds from ES were identified by UPLC-QTOF-MS/MS, 47 compounds were deduced by secondary mass spectrometry information, and 20 compounds were confirmed by comparing with the reference substance. The total ion chromatogram (TIC) is shown in Figure 1.

**Figure 2:**
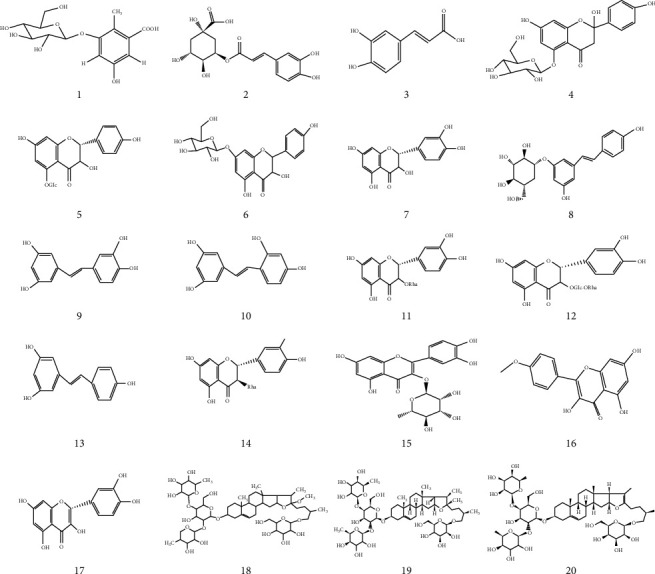
The structures of 20 compounds.

The UPLC-QTOF-MS/MS information and structures of 20 compounds are shown in Figure 2 and Table 3, respectively.

### 3.2. *In Vivo* Anti-Inflammatory Activity

**Figure 3:**
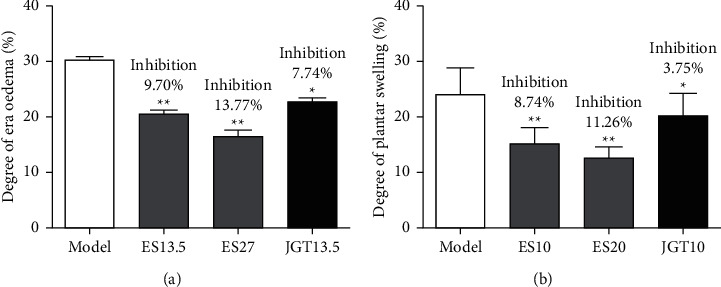
In vivo anti-inflammatory activity of ES. ES and JGT capsule inhibited xylene-induced ear oedema in mice (a) and egg white-induced plantar swelling in rats (b). Saline (model group), ES (the extract at the doses equalling to 13.5 and 27 g/kg in mice, 10 and 20 g/kg in rats), and JGT (the extract at the dose equalling to 13.5 g/kg in mice, 10 g/kg in rats) were orally administered, respectively. The mouse ear oedema and rat plantar swelling were induced, and the inhibitory activities were calculated as described in Materials and Methods, respectively. Data are presented as means ± SD (*n* = 10). ^∗^*P* < 0.05 and ^∗∗^*P* < 0.01 versus the model group.

Applying of xylene-induced ear oedema in mice (Figure 3(a)), indicating acute inflammation. ES (the extract at the doses equalling to 13.5 and 27 g herb/kg) and JGT (the extract at the dose equalling to 13.5 g herb/kg) inhibited ear oedema with inhibiting rates of 9.70, 13.77, and 7.74%, respectively (Figure 3(a)).

Subcutaneous injection of egg white aqueous solution induced plantar swelling in rats (Figure 3(b)), indicating acute inflammation. ES (the extract at the doses equalling to 10 and 20 g herb/kg) and JGT (the extract at the dose equalling to 10 g herb/kg) inhibited plantar swelling with inhibiting rates of 8.74, 11.26, and 3.75%, respectively (Figure 3(b)).

### 3.3. Cytotoxicity of ES on THP-1 Cells

**Figure 4:**
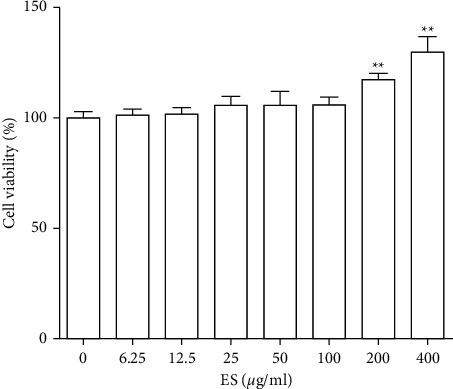
Effect of ES on the viability of THP-1 cells. Cells were treated with various concentrations (0, 6.25, 12.5, 25, 50, 100, 200, and 400 *μ*g/mL) for 24 h and their viability was determined using a Cell Counting Kit (CCK-8). Data are presented as means ± SD (*n* = 3). ^∗∗^*P* < 0.01 versus the blank control group.

The effects of ES on THP-1 growth were examined by CCK-8 assay, and no cytotoxic effect was observed at 100 *μ*g/mL concentration, while there was significant proliferation when the cells were treated with 200 and 400 *μ*g/mL ES (Figure 4). Therefore, sample treatments between 6.25 and 100 *μ*g/mL were used in the subsequent experiments.

### 3.4. Selective Inhibitory Activity of COX-2

**Figure 5:**
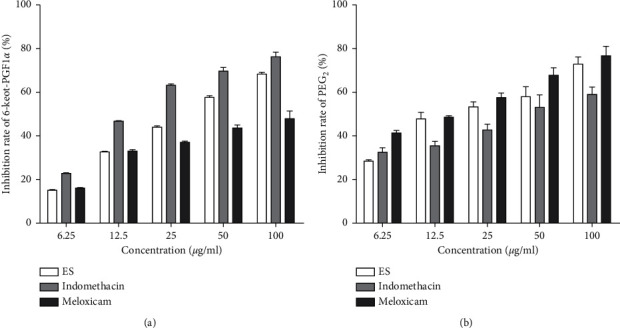
Effects of ES, indomethacin, and meloxicam on 6-Keot-PGF1*α* (a) and PGE2 (b) production in LPS-induced THP-1 cells. Cells were treated with ES, indomethacin, and meloxicam (6.25, 12.5, 25, 50, and 100 *μ*g/mL) for 12 h after exposure to LPS (1 *μ*g/mL) for 12 h. The supernatant was collected to ELISA kits. Data are presented as means ± SD (*n* = 3).

The PGE_2_ produced in LPS-induced THP-1 cells was quantified to determine COX-2 activity using a PGE_2_ standard curve, while the 6-Keot-PGF_1_*α* was quantified to determine COX-1 activity. The inhibitory activity of 6-Keot-PGF_1_*α* and PGE_2_ was expressed by the inhibition rate (Figure 5). IC_50_ was calculated, and IC_50_ (COX-1)/IC_50_ (COX-2) showed the selective inhibitory activity of COX-2.

**Figure 6:**
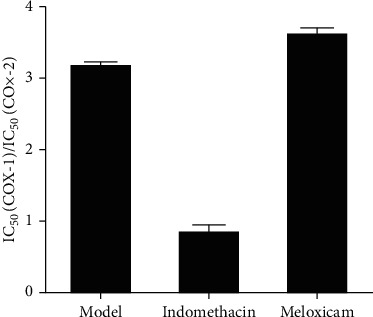
IC_50_ (COX-1)/IC_50_ (COX-2) of three drugs.

Treatment with ES reduced the expression of 6-Keot-PGF_1_*α* and PGE_2_ in a dose-dependent manner (Figure 5). Moreover, it showed that IC_50_ (COX-1)/IC_50_ (COX-2) of ES was 3.15, which was greater than 1 and close to the IC_50_ (COX-1)/IC_50_ (COX-2) of meloxicam (Figure 6 and Table 4). These results implied that ES had selective COX-2 inhibitory activity.

### 3.5. Effect of ES on the Expressions of Proinflammatory Cytokines IL-1*β*, IL-6, and TNF-*α*

**Figure 7:**
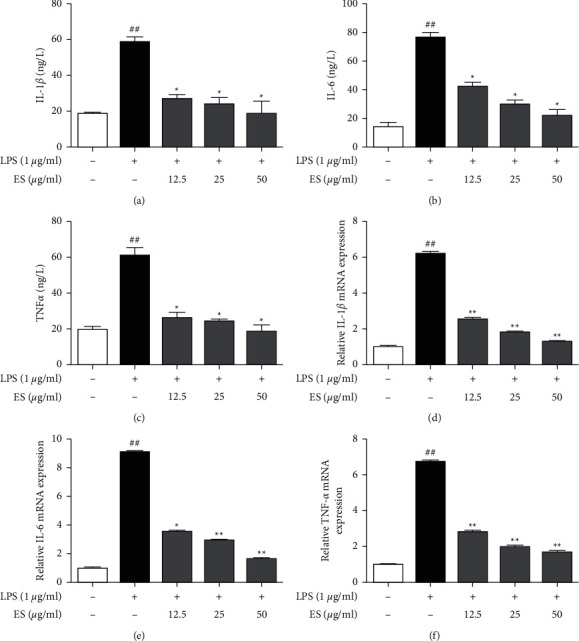
Effect of ES on proinflammatory cytokine production and gene expression in LPS-induced THP-1 cells. Cells were treated with LPS (1 *μ*g/mL) and ES (12.5, 25, 50 *μ*g/mL) for 12 h. The IL-1*β*, IL-6, and TNF-*α* contents were measured using ELISA kits (a–c). The mRNA expression levels of IL-1*β*, IL-6, and TNF-*α* were determined by qRT-PCR and normalized to the *β*-actin levels (d–f). Data are presented as means ± SD (*n* = 3). ^##^*P* < 0.01 versus the blank control group. ^∗^*P* < 0.05 and ^∗∗^*P* < 0.01 versus the LPS-treated group.

ES dose-dependently decreased LPS-induced expression of IL-1*β*, IL-6, and TNF-*α*. Treatment with ES at the 12.5 *μ*g/mL significantly decreased IL-1*β*, IL-6, and TNF-*α* to 27.25 ± 2.17 ng/L, 42.54 ± 2.68 ng/L, and 26.14 ± 2.93 ng/L, respectively (Figures 7(a)–7(c)). At the 50 *μ*g/mL concentration of ES, the expressions of IL-1*β*, IL-6, and TNF-*α* were basically the same as those in the blank group. Likewise, ES significantly inhibited the expressions of IL-1*β*, IL-6, and TNF-*α* mRNA (Figures 7(d)–7(f)).

### 3.6. Effect of ES on MAPK Signaling Pathway in LPS-Induced THP-1 Cells

**Figure 8:**
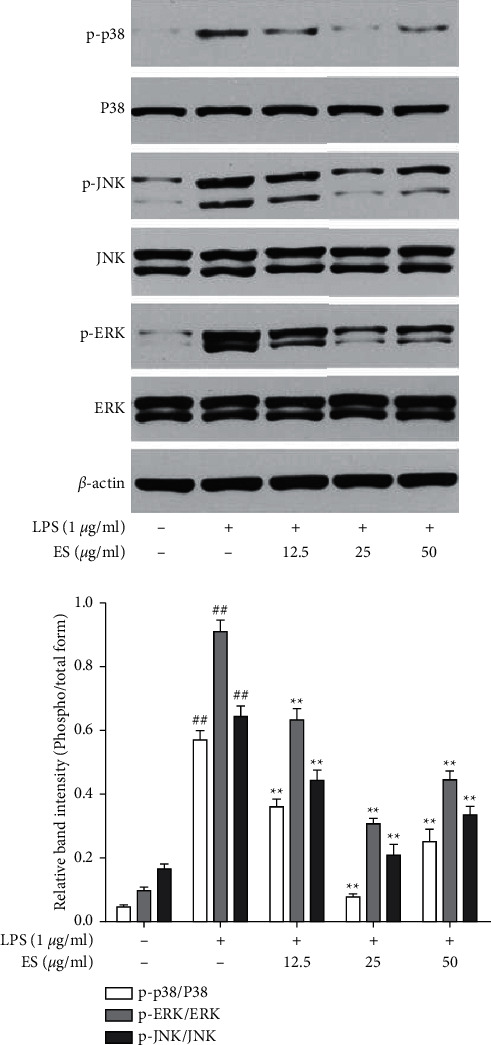
Effect of ES on MAPK signaling pathway in LPS-induced THP-1 cells. Cells were treated with ES (12.5, 25, and 50 *μ*g/mL) for 1 h and then incubated with or without LPS (1 *μ*g/mL) for 1 h. Protein expression was tested by western blot (^##^*P* < 0.01 versus the blank control group; ^∗^*P* < 0.05 and ^∗∗^*P* < 0.01 versus the LPS-treated group).

As presented in Figure 8, the phosphorylation levels of p38, JNK, and ERK1/2 were increased in THP-1 cells treated with LPS alone, and ES decreased LPS-induced phosphorylation of p38, JNK, and ERK1/2.

### 3.7. Effect of ES on NF-*κ*B Signaling Pathway in LPS-Induced THP-1 Cells

**Figure 9:**
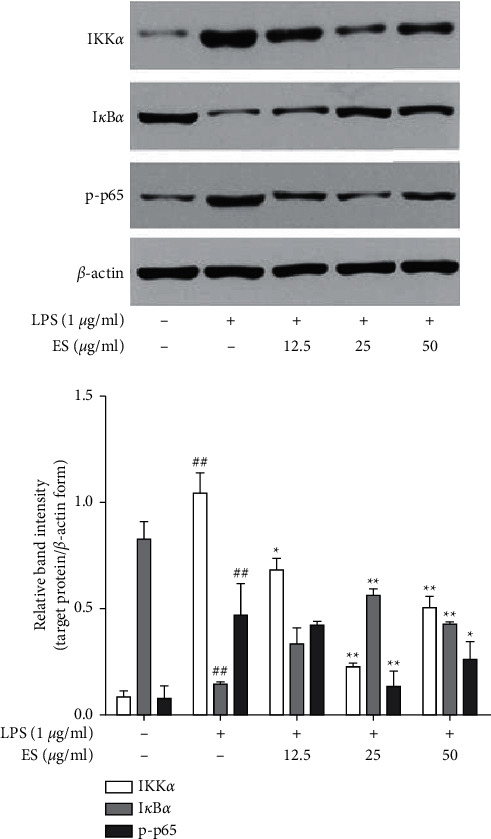
Effect of ES on the NF-*κ*B signaling pathway in LPS-induced THP-1 cells. Cells were treated with ES (12.5, 25, and 50 *μ*g/mL) for 1 h then incubated with or without LPS (1 *μ*g/mL) for 1 h. Protein expression was tested by Western blot (^##^*P* < 0.01 versus the blank control group; ^∗^*P* < 0.05 and ^∗∗^*P* < 0.01 versus the LPS-treated group).

To further explore the inhibiting effect of ES, its effect on NF-*κ*B signaling pathway was investigated. The expression levels of IKK*α*, I*κ*B*α*, and p-p65 were evaluated by Western blotting. As seen in Figure 9, ES suppressed LPS-induced expression of IKK*α* and degradation of I*κ*B*α* and further inhibited phosphorylation of p65.

## 4. Discussion

The extract from *Smilax china* L. has been confirmed to have anti-inflammatory activities, which are likely related to flavonoids, saponins, and polyphenols. In this study, the activities of ES in equal doses were significantly better than the positive control “Jin Gang Teng” capsule in the *in vivo* acute inflammatory model. Furthermore, ES selectively inhibited LPS-induced overrelease of COX-2, as well as suppressed the transcription and expression of IL-1*β*, IL-6, and TNF-*α* in THP-1 cells. ES exerted this anti-inflammatory effect through negative regulating MAPK and NF-*κ*B pathways.

Prostaglandins are important mediators of the body's response to pain and inflammation and are formed from essential fatty acids found in cell membranes. This reaction is catalysed by cyclooxygenase, a membrane-associated enzyme occurring in two isoforms: COX-1 and COX-2 [17]. The physiological prostaglandins are produced by catalytic COX-1, represented by PGI_2_, and PGI_2_ is very unstable in the body, which will be metabolized to stable 6-Keot-PGF_1_*α*. Therefore, the secretion of 6-Keot-PGF_1_*α* can reflect the activity of COX-1. The pathological prostaglandins are produced by catalytic COX-2, represented by PGE_2_, so the secretion of PGE_2_ can reflect the activity of COX-2 [18].

COX-2 is closely related to inflammation and its product PGE_2_ is an important inflammatory medium [19]. Classic epoxy enzyme inhibitors such as aspirin, indomethacin, and many other nonsteroidal anti-inflammatory drugs (NSAIDs) inhibit both COX-1 and COX-2, which play anti-inflammatory effect accompanied by gastrointestinal and renal side effects [20]. COX-2 selective inhibitors such as meloxicam have a weak inhibitory activity of COX-1, consequently reducing the adverse reactions.

ES inhibited overproduction of 6-Keot-PGF_1_*α* and PGE_2_ induced by LPS, and the inhibition rate of PGE_2_ was greater than 6-Keot-PGF_1_*α* at the same concentration (Figure 5). Moreover, it showed that IC_50_ (COX-1)/IC_50_ (COX-2) of ES was 3.15, which was greater than 1 and close to the IC_50_ (COX-1)/IC_50_ (COX-2) of meloxicam (Figure 6). These results suggested that ES had selective COX-2 inhibitory activity; therefore, ES may be used as a potentially developable drug for inflammation diseases.

Proinflammatory cytokines such as IL-1*β*, IL-6, and TNF-*α*, which play crucial roles in the development of inflammatory diseases, are also involved in the innate immunity and autoimmune diseases [21]. Thus, blocking the effects of proinflammatory mediators offers an attractive therapeutic strategy. In this study, ES suppressed LPS-induced overproduction of IL-1*β*, IL-6, and TNF-*α* in a dose-dependent manner (Figures 7(a)–7(c)).

In THP-1 cells, LPS induces IL-1*β*, IL-6, and TNF-*α* transcription leading to overproduction of IL-1*β*, IL-6, and TNF-*α*; thus, the effects of ES on the expression of IL-1*β*, IL-6, and TNF-*α* mRNAs were further examined [22]. The present study revealed that ES significantly and dose-dependently suppressed the expression of IL-1*β*, IL-6, and TNF-*α* mRNAs in LPS-induced THP-1 cells (Figures 7(d)–7(f)). These results indicated that ES inhibited inflammation by regulating the gene transcription of proinflammatory cytokines.

Activation of MAPKs is known to be associated with the expression of multiple genes that together regulate the inflammatory processes. MAPK family with three major components p38, JNK, and ERK has been reported to play an important role in the upregulation of biosynthesis of IL-1*β*, IL-6, and TNF-*α* and transcriptional target enzymes (e.g., COX-2 and iNOS) [23]. Therefore, the effects of ES on LPS-induced phosphorylation of MAPKs in THP-1 cells were evaluated using Western blot analysis. As shown in Figure 8, the phosphorylation levels of p38, JNK, and ERK were increased in cells treated with LPS alone. Importantly, ES treatment significantly inhibited phosphorylation of p38, JNK, and ERK at all concentrations from 12.5 *μ*g/mL to 50 *μ*g/mL. These results revealed that the high activity of ES against the LPS-induced inflammatory stimuli was attributed to the inhibition of the phosphorylation of p38, JNK, and ERK, which may downregulate the levels of inflammatory mediators and cytokines in THP-1 cells. Interestingly, ES exhibited high activity at 25 *μ*g/mL, a much better inhibition potential in comparison to the concentration of 50 *μ*g/mL. The more pronounced effect at intermediate concentration than high concentration may be due to the multicomponent and multitarget synergistic effect, which is very common in traditional Chinese medicines [24, 25].

During the immune cells rest stage, NF-*κ*B remains inactive as part of a complex with p65, p50, and I*κ*B*α*. I*κ*B*α* is an inhibitory protein that binds to the p50/p65 heterodimer in the cytoplasm. Upon stimulation by LPS or certain cytokines, I*κ*B*α* is phosphorylated by the I*κ*B kinase (IKK) and degraded, promoting the activation and phosphorylation of p65 subunit that allows the translocation of NF-*κ*B into the nucleus and leads to the transcription of proinflammatory mediators [26]. Thus, the underlying inhibitory mechanism was then focused on the LPS-stimulated cellular NF-*κ*B pathway and changes in the content and forms of IKK, I*κ*B-*α*, and p65 were investigated. Western blot analysis indicated that NF-*κ*B pathway was activated upon the addition of LPS with significant overexpression of IKK*α* and phosphorylated p65 protein as well as degradation of I*κ*B-*α* (Figure 9). Moreover, there was observable inhibition of IKK*α* overproduction by ES at three concentrations. Simultaneously, ES treatment increased level of I*κ*B-*α* and decreasd phosphorylated p65 (Figure 9). Because activation of IKK allows I*κ*B*α* to be phosphorylated and ubiquitinated from p50/p65 complex, which leads to phosphorylation of p65, it is likely that ES downregulated expression of IKK*α* that resulted in the prevention of the degradation of I*κ*B-*α* and phosphorylation of p65. In addition, this was consistent with the results of LPS-stimulated cellular MAPK pathway showing that ES was more effective at intermediate concentration on NF-*κ*B regulated proteins. All these results implied that ES blocked the activation of the NF-*κ*B pathway in LPS-induced THP-1 cells through inhibition of IKK*α* overexpression.

Based on UPLC-QTOF-MS/MS, 20 compounds from ES were explored and verified by the reference substance, which mainly were flavonoids, saponins, and tannins including taxifolin, astilbin, rutin, resveratrol, polydatin, oxyresveratrol, engeletin, quercitrin, quercetin, and methylprotodioscin. Many flavonoids, saponins, and tannins have been shown to participate in the regulation of inflammatory mediators and signal transduction pathways [27–29]. For example, resveratrol suppressed the expression of TNF-*α*, IL-6, and COX-2 through a decrease in the intracellular levels of ERK1/2, as well as activation of NF-*κ*B in activated HMC-1 cells [30]. Polydatin effectively inhibited NO and PGE_2_ production and reduced iNOS and COX-2 expression at protein and transcriptional levels through NF-*κ*B and MAPK pathways in LPS-induced RAW264.7 cells [31]. However, some compounds play an anti-inflammatory role by regulating specific targets. In previous studies, taxifolin suppressed expression of IL-1*β* and IL-6 mRNA in LPS-activated RAW264.7 cells, but not that of TNF-*α* [32]. Astilbin significantly suppressed production of NO and TNF-*α*, as well as mRNA expression of iNOS and TNF-*α* in LPS-induced RAW 264.7 cells, but did not affect IL-6 release or its mRNA expression [33]. Oxyresveratrol inhibited iNOS expression rather than iNOS enzyme activity through downregulation of NF-*κ*B binding activity and significant inhibition of COX-2 activity [34]. Methylprotodioscin inhibited the activation of JNK and c-Jun while the activation of p38 MAPK, ERK, and NF-*κ*B was not significantly affected [35]. Rutin protected spinal cord cells by reducing oxidative stress and inflammation and by decreasing the expression of proapoptotic proteins via inhibition of the p38 MAPK pathway [36]. The composition of ES is complex, and the targets and effects of each component are different. Thus, the anti-inflammatory effect of ES is likely achieved by multiple components and multiple targets.

## 5. Conclusions

**Figure 10:**
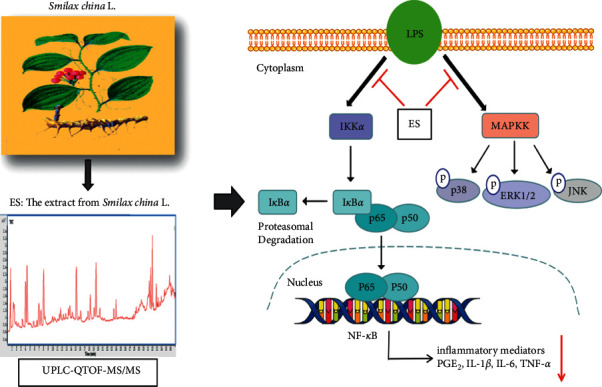
Graphical abstract. The ES was prepared using ethanol extraction and macroporous adsorption resin purification. The components of ES were identified by UPLC-QTOF-MS/MS. The anti-inflammatory effect of ES was associated with the inhibition of IL-1*β*, IL-6, and TNF-*α* via negative regulation of MAPK and NF-*κ*B signaling pathways in LPS-induced THP-1 cells.

In conclusion, ES prepared using ethanol extraction and macroporous adsorption resin purification mainly contained flavonoids, saponins, and tannins, and 20 compounds from ES were confirmed by comparing with the reference substance. ES inhibited the overproduction of 6-Keot-PGF_1_*α* and PGE2 induced by LPS and showed selective COX-2 inhibitory activity. ES decreased production and mRNA levels of proinflammatory cytokines (IL-1*β*, IL-6, and TNF-*α*). ES also blocked the activation of the MAPK and NF-*κ*B pathways, which may be closely related to the inhibitory effects of ES on inflammatory mediators (Figure 10). Moreover, ES may exert anti-inflammatory effect through multiple components and multiple targets.

## Figures and Tables

**Table 1:** The elution program.

*t*/min	A : B
0 ⟶ 5	12 : 88 ⟶ 15 : 85
5 ⟶ 12	15 : 85 ⟶ 24 : 76
12 ⟶ 15	24 : 76
15 ⟶ 18	24 : 76 ⟶ 30 : 70
18 ⟶ 20	30 : 70
20 ⟶ 26	30 : 70 ⟶ 42 : 58
26 ⟶ 28	42 : 58 ⟶ 60 : 40
28 ⟶ 35	60 : 40 ⟶ 5 : 95
35 ⟶ 37	5 : 95

**Table 2:** qRT-PCR primer sequences.

Gene	Forward primer	Reverse primer
IL-1*β*	5′-AAACAGATGAAGTGCTCCTTCCAGG-3′	5′-TGGAAGAACACCACTTGTTGCTCCA-3′
IL-6	5′-AGTGAGGAACAAGCCAGAGC-3′	5′-GCATTTGTGGTTGGGTCAG-3′
TNF-*α*	5′-TTCCTCAGCCTCTTCTCCTT-3′	5′-GCTACAGGCTTGTCACTCGG-3′
GAPDH	5′-CCATGTTCGTCATGGGTGTGAACCA-3′	5′-GCCAGTAGAGGCAGGGATGATGTTC-3′

**Table 3:** UPLC-QTOF-MS/MS information of 20 compounds.

No.	*t*/min	[M-H]-1 (m/z)	Fragment ion (m/z)	Formula	Name
1	4.004	329.0896	269, 191, 167	C_14_H_18_O_9_	2-Methyl-5-hydroxybenzoic acid-3-O-*β*-D-Glucoside
2	4.378	353.0908	191, 161, 241	C_16_H_18_O	Chlorogenic acid
3	4.920	179.0350	171, 135	C_9_H_8_O_4_	Caffeic acid
4	5.827	449.1109	287, 259, 153	C_21_H_22_O_11_	2,7,4-Trihydroxydihydroflavone-5-O-*β*-D-glucoside
5	6.845	449.1124	287, 269, 153	C_21_H_22_O_11_	7,4-Dihydroxydihydroflavonol-5-O-*β*-D-glucoside
6	8.041	449.1138	287, 269, 259	C_21_H_22_O_11_	7,4′-Dihydroxydihydroflavonol-7-O-*β*-D-glucoside
7	11.076	303.0528	285, 177, 153	C_15_H_12_O_7_	Taxifolin
8	11.806	389.1274	242, 227	C_20_H_22_O_8_	Polydatin
9	12.806	243.0671	174, 159, 130	C_14_H_12_O_4_	Piceatannol
10	13.689	243.0672	201, 174, 130	C_14_H_12_O_4_	Oxyresveratrol
11	15.248	449.1119	303, 285, 241	C_21_H_22_O_11_	Astilbin
12	17.284	609.1505	484, 301, 220	C_27_H_30_O_16_	Rutin
13	17.759	227.0718	185, 164, 143	C_14_H_12_O_3_	Resveratrol
14	19.438	433.1172	369, 269, 225	C_21_H_22_O_10_	Engeletin
15	20.354	447.0956	301, 255, 179	C_21_H_21_O_11_	Quercitrin
16	28.393	285.0408	218, 195, 151	C_15_H_10_O_6_	Kaempferide
17	30.886	301.0359	254, 186, 118	C_15_H_10_O_7_	Quercetin
18	30.970	1061.5500	1107, 915, 641	C_52_H_86_O_22_	Methylprotodioscin
19	31.021	1047.5370	1093, 901, 597	C_51_H_84_O_22_	Protodioscin
20	31.928	1029.5290	1075, 541, 883	C_51_H_82_O_21_	Pseudoprotodioscin

**Table 4:** Selective COX-2 inhibitory activity of three drugs.

Medicine	IC_50_ (COX-1) (mg/L)	IC_50_ (COX-2) (mg/L)	IC_50_ (COX-1)/IC_50_ (COX-2)
ES	77.95	24.74	3.15
Indomethacin	37.86	45.91	0.82
Meloxicam	136.57	37.93	3.60

## Data Availability

The data used to support the findings of this study are available from the corresponding author upon request.

## Abbreviations

ES: The extract from *Smilax china* L.
LPS: Lipopolysaccharide
UPLC-QTOF-MS/MS: Ultra-performance liquid chromatography coupled with quadrupole time-of-flight tandem mass spectrometry
JGT: Jin Gang Teng
COX-2: Cyclooxygenase-2
PGE_2_: Prostaglandin E2
NF-*κ*B: Nuclear factor kappa-B
MAPKs: Mitogen-activated protein kinases
ERK: Extracellular signal-regulated kinase
JNK: C-jun N-terminal kinase
IL-1*β*: Interleukin-1*β*
IL-6: Interleukin-6
TNF-*α*: Tumor necrosis factor
IC_50_: The half-maximal inhibitory concentration
TIC: Total ion chromatogram
PGI_2_: Prostaglandin I2.
